# The Diverse Mechanisms of miRNAs and lncRNAs in the Maintenance of Liver Cancer Stem Cells

**DOI:** 10.1155/2018/8686027

**Published:** 2018-05-15

**Authors:** Jing Zhao, Yan Fu, Jing Wu, Juan Li, Guangjian Huang, Lunxiu Qin

**Affiliations:** ^1^Department of General Surgery, Huashan Hospital, Fudan University, Shanghai 200040, China; ^2^Cancer Metastasis Institute, Fudan University, Shanghai 200040, China; ^3^Department of Infectious Diseases, Huashan Hospital, Fudan University, Shanghai 200040, China

## Abstract

Liver cancer is the second leading cause of cancer-related death worldwide. The high frequency of recurrence and metastasis is the main reason for poor prognosis. Liver cancer stem cells (CSCs) have unlimited self-renewal, differentiation, and tumor-regenerating capacities. The maintenance of CSCs may account for the refractory features of liver cancer. Despite extensive investigations, the underlying regulatory mechanisms of liver CSCs remain elusive. miRNA and lncRNA, two major classes of the ncRNA family, can exert important roles in various biological processes, and their diverse regulatory mechanisms in CSC maintenance have acquired increasing attention. However, to the best of our knowledge, there is a lack of reviews summarizing these findings. Therefore, we systematically recapitulated the latest studies on miRNAs and lncRNAs in sustaining liver CSCs. Moreover, we highlighted the potential clinical application of these dysregulated ncRNAs as novel diagnostic and prognostic biomarkers and therapeutic targets. This review not only sheds new light to fully understand liver CSCs but also provides valuable clues on targeting ncRNAs to block or eradicate CSCs in cancer treatment.

## 1. Introduction

Liver cancer is one of the most common malignancies and is ranked as the second leading cause of cancer-related death around the world [1]. Despite great progress in prevention, diagnosis, and treatment, the prognosis of liver cancer remains dismal due to frequent recurrence and metastasis [2]. Similar to most malignancies, liver cancer is composed of a heterogeneous cell hierarchy, in which a distinct small subset referred to as cancer stem cells (CSCs) resides [3]. CSCs have unlimited proliferation, self-renewal, differentiation, and tumor-regenerating capacities, which lead to tumor initiation, relapse, metastasis, and drug resistance [4]. Liver CSCs are the main obstacle to the cure for refractory liver cancer. Recently, several surface markers have been widely used to isolate liver CSCs, including CD13, CD133, CD24, EpCAM, CD44, CD90, and OV6. In addition, the normal stemness-related transcriptional factors and developmental signaling pathways also exert critical roles in the maintenance of CSCs, such as the AKT, Wnt/*β*-catenin, and IL-6/STAT3 cascades [5]. Though numerous coding genes are reported to be involved in liver CSCs, these regulatory mechanisms are largely inadequate for the complete understanding and eradication of CSCs.

With the development of whole genome and transcriptome sequencing technologies, numerous noncoding RNAs (ncRNAs) without protein-coding potential have been identified [6]. ncRNAs are no longer treated as “evolutionary junk.” Increasing evidence demonstrates that ncRNAs play important regulatory roles in various biological processes, including cancer development [7–11]. According to the relative length of nucleotides, ncRNAs are briefly classified into two categories: small or short RNA with lengths less than 200 nucleotides and long noncoding RNA (lncRNA), which is longer than 200 nucleotides in length. miRNA, as the representative member of small RNAs, has been well studied [12]. Through imperfectly binding to the 3′-untranslated region (3′-UTR) of target mRNAs, miRNA can induce mRNA degradation or inhibit translation to silence target gene expression [13]. Unlike miRNA, lncRNA has extremely complicated functions and mechanisms to manipulate gene expression in cis- and in trans-manners. LncRNA can interact with DNA, RNA, and proteins to affect transcriptional machinery assembly, alternative splicing processes, and chromatin remodeling [14]. Dysregulated expression of miRNAs and lncRNAs has been found in multiple cancers, and these aberrant ncRNAs have oncogenic or tumor suppressive functions as well as acting as coding genes [15]. Accumulating investigations have demonstrated that miRNAs and lncRNAs are essential for sustaining CSC properties in liver cancer, like miR-200b, miR-181, miR-1246, lncTCF-7, lnc-DANCR, lnc-PVT1, and so on [16–21]. These findings provide new insights regarding the complexity of liver CSCs and help foster an understanding of the underlying mechanisms. In this review, we systematically summarize the latest findings of miRNAs and lncRNAs on liver CSCs and illustrate their diverse mechanisms. Accordingly, we may develop ncRNA-based novel approaches to conquer CSCs in the future.

## 2. The Underlying Mechanisms of miRNAs in the Regulation of Liver CSCs

MiRNAs, with a length of 19~25 nucleotides, usually function as negative regulators to repress gene expression via base-pair complementation with the 3′-UTR of target genes. Currently, many miRNAs' alterations have been documented to participate in liver CSC regulation via various targets, as listed in Table 1. We summarize the detailed mechanisms as follows.

### 2.1. miRNAs Regulate Liver CSCs by Affecting the Wnt/*β*-Catenin Cascade

The Wnt/*β*-catenin signaling pathway has been shown to play an important role in regulating stem cell and tumorigenic properties in liver cancer. Inhibition of the Wnt pathway has also been shown to be effective in eliminating CSC-like features [22, 23]. When a Wnt ligand binds to its receptor FZD or LRPs, cytoplasmic Dvl is phosphorylated, which dissociates *β*-catenin from the Axin/APC/GSK3*β* destructive complex. Then, *β*-catenin accumulates and is translocated into the nucleus to form the *β*-catenin/LEF/TCF transcriptional complex, which initiates the transcription of downstream genes to regulate liver CSC maintenance [24]. Several miRNAs are reported to affect the Wnt/*β*-catenin cascade to regulate liver CSCs.

The miR-181 family is critical for maintaining the “stemness” of EpCAM^+^ liver CSCs. These miRNAs can directly bind to the 3′-UTR of CDX2, GATA6, and NLK mRNAs and inhibit their expression. CDX2 and GATA6 are the transcriptional regulators for hepatic differentiation, and NLK functions as the inhibitor of Wnt/*β*-catenin signaling. Therefore, miR-181 can enhance the self-renewal ability of EpCAM^+^ liver CSCs and maintain the undifferentiated state simultaneously [17]. In addition, miR-1246 can silence AXIN2 and GSK3*β* expression, leading to the nuclear accumulation and activation of *β*-catenin, which promotes stem cell-like phenotypes in CD133^+^ liver CSCs. Moreover, the important CSC transcriptional factor OCT4 can directly bind to the miR-1246 promoter to increase its expression [18]. Enhancing let7b has also been reported to decrease the ratio of CD24^+^133^+^ in liver cancer cells via inhibiting FZD4 expression to inactivate the Wnt/*β*-catenin signaling cascade [25]. Li et al. defined the miR-148a-ACVR/BMP circuit as having a regulatory role in liver CSCs. ACVR1 is an important receptor of the bone morphogenetic protein (BMP) that is closely implicated in the regulation of BMP/Wnt signaling. These authors found that miR-148a can inhibit the expression of ACVR1 by binding to the 3′-UTR of ACVR1, further leading to the downregulation of the direct downstream targets of the Wnt signaling pathway [26, 27]. There are also several other miRNAs that are reported to inhibit the stemness of liver CSCs through the Wnt/*β*-catenin pathway. For example, miRNA-200a can directly repress *β*-catenin; miRNA-214 can directly target *β*-catenin and indirectly inhibit *β*-catenin through EZH2 together; miR-612 can indirectly decrease the nuclear accumulation of *β*-catenin, and let-7a can deplete TCF4 [28–31].

### 2.2. miRNAs Regulate Liver CSCs by Affecting the PTEN/PI3K/AKT/Bad Signaling Pathway

PTEN is a well-known tumor suppressor that serves as the natural inhibitor of PI3K to negatively regulate AKT. MiR-25 can directly target PTEN to stimulate the PI3K/AKT pathway, which enables liver CSCs to resist apoptosis [32]. MiR-21 is upregulated in liver CSCs and can promote the migration and invasion of liver CSCs. A mechanistic study revealed that miR-21 can target the tumor suppressors PTEN, RECK, and PDCD4 to reduce their protein expression without affecting the mRNA levels [33].

### 2.3. miRNAs Affect Liver CSC Maintenance via Stemness-Related Transcriptional Factors and Markers

miR-612 can target and decrease the expression of SP1, which is an important transcriptional activator of the stemness-factor, Nanog. Through silencing SP1 to reduce Nanog expression, miR-612 can shrink the number and size of liver CSCs [34]. OCT4 is another critical transcriptional factor for the maintenance of stem cells and liver CSCs [57, 58]. Li et al. reported that the overexpression of miR-429 endows EpCAM^+^ liver CSCs to increase stem cell-associated gene expression, self-renewal, chemotherapeutic resistance, and tumorigenicity capacities. They found that miR-429 can inhibit the expression of PBBP4 by binding to its 3′-UTR, which promotes the transcription activity of E2F1 on OCT4. Successively, the increased OCT4 is recruited to the EpCAM promoter and enhances its expression to strengthen liver CSCs' properties [35]. It was also reported that miR-145 participates in the regulation of the stemness of liver CSCs through direct modulation of OCT4 [36]. Surface markers, such as CD13 and CD24, are functionally implicated in tumor development and progression. CD13 can help HCC CSCs achieve resistance to chemotherapy by inducing cells into dormancy and decreasing the accumulation of reactive oxygen species, DNA damage, and cell death [59, 60]. CD24 can help to activate the STAT3 signal, subsequently inducing Nanog expression to sustain CSC traits [61]. miR-200b is found to inhibit liver CSC formation via two independent mechanisms. On the one hand, miR-200b can directly suppress BMI1 expression, a stemness-related transcriptional factor. On the other hand, miR-200b can directly target ZEB1, which acts as a transcriptional activator to promote CD13 and CD24 expression [16]. CD133 is another type of surface marker that plays an important role in maintaining liver tumorigenesis [62]. miR-142-3p can bind to the 3′-UTR of CD133 and inhibit its expression, thereby attenuating the stemness of CD133^+^ liver CSCs [37]. Han et al. found that *α*2*δ*1 may serve as a novel liver CSC marker. miR-424, miR-222, miR-200b, and let-7c are downregulated in *α*2*δ*1^+^ liver CSCs, which synergistically play important roles in the acquisition and maintenance of liver CSC properties. These researchers found that low expression levels of the four miRNAs can increase the expression of PBX3, which can activate critical genes for liver CSCs, including CACNA2D1, EpCAM, SOX2, SALL2, NOTCH3, and WNT10A [38].

### 2.4. miRNAs Regulate Liver CSCs through Metabolic Reprogramming

Metabolic reprogramming is one of the most common cancer hallmarks. For example, the famous “Warburg effect” indicates that cancer cells prefer to elevate glycolysis and lactate production even in the presence of oxygen [63]. Song et al. reported that enhanced glycolysis is associated with CD133^+^ liver CSC characteristics, and the downregulation of miR-122 plays an important role in the abnormal metabolic process. Downregulation of miR-122 leads to the upregulated expression of its direct target, pyruvate dehydrogenase kinase 4 (PDK4). PDK4 can stimulate glycolysis and further increase the stemness gene expression and spheroid formation capacity in CD133^+^ liver CSCs [39].

### 2.5. miRNAs Regulate Liver CSCs by Affecting Tumor-Associated Genes

TP53INP1 is a tumor suppressor and has antiproliferative and proapoptotic activities [64, 65]. miR-130b, upregulated in CD133^+^ liver CSCs, can repress TP53INP1 expression by directly targeting its 3′-UTR of TP53INP1, which helps CSCs to enhance proliferation, resist the chemotherapeutic drug, doxorubicin, and increase the expression of a series of stem cell-associated genes, including *β*-catenin, Notch-1, and Nestin [40]. miR-155 is also reported to regulate liver CSCs via targeting TP53INP1 [41]. KIT, a well-established oncogene, can be suppressed by miR-152 directly by binding to the 3′-UTR of KIT, thus inhibiting cell proliferation and colony formation of CD133^+^ liver CSCs [42]. MAP3K8 is a well-known oncogene in various human tumors. miR-589-5p can target MAP3K8 and decrease its expression to suppress stemness-associated genes, including Oct4, Sox2, and Nanog, thereby reducing the capacity of forming self-renewal spheres and tumorigenicity [43]. miR-150 is able to target the oncogene C-Myb, by which miR-150 can inhibit C-Myb downstream genes, such as cyclin D1 and Bcl-2, to induce cell cycle arrest and apoptosis in CD133^+^ cells [44]. miR-148b is downregulated in liver CSCs, which can repress the oncogene NRP1 to inhibit proliferation, metastasis, tumorigenesis, and drug resistance in liver CSCs [45]. miR-137 has a tumor suppressive role and can target the adenine nucleotide translocator ANT2. The downregulation of miR-137 enhances the sphere-forming ability and resistance to sorafenib therapy as well as increasing CD133, CD44, and EpCAM expression, which accounts for the phenotypes of the liver CSCs [46]. TGF-*β* is an antimitogenic cytokine that becomes oncogenic in advanced tumors [66]. The restoration of miR-122 has been reported to be able to induce a dormant state of stem-like HCC through the Smad-independent TGF-*β* pathway [67].

## 3. The Underlying Mechanisms of lncRNA in the Regulation of Liver CSCs

In contrast to miRNAs, lncRNAs have versatile mechanisms to control gene expression at both the transcriptional and posttranscriptional levels. lncRNAs can serve as a scaffold to recruit transcriptional factors within the promoter region to affect gene expression. lncRNAs can directly bind to DNA and RNA via a complementary sequence to impact transcriptional initiation or RNA stability. Additionally, lncRNA can modulate the posttranslational modification of proteins. Numerous lncRNAs have been demonstrated to be deregulated in cancers and exert critical roles in cancer development, such as malignant proliferation, metastasis, invasion, antiapoptosis, therapeutic resistance, and CSC formation. Recently, accumulating studies have focused on the regulation of lncRNAs in liver CSCs, as listed in Table 2. The underlying mechanisms are addressed as follows.

### 3.1. lncRNAs Sustain Liver CSCs by Activating the Wnt/*β*-Catenin Pathway

CD133 and CD13 are two widely used liver CSC surface markers, and the double-positive cell fraction exhibits obvious CSC properties, including a strong self-renewal capacity and chemical drug resistance. The transcriptome microarray analysis identified many differentially expressed lncRNAs in the CD13^+^ CD133^+^ cell population. Among these dysregulated lncRNAs, lncTCF7 and lnc-*β*-Catm are the most upregulated lncRNAs, which play critical roles in driving CSC self-renewal and tumor propagation via activating the Wnt/*β*-catenin pathway [19, 47]. lncTCF7 is located at chromosome 5, the neighboring TCF7 gene. lncTCF7 directly interacts with the SWI/SNF complex, and the evolutionally conserved SWI/SNF complex can hydrolyze ATP to provide energy for mobilizing nucleosomes and remodeling chromatin. Through the association, lncTCF7 recruits the SWI/SNF to the promoter region of TCF7 and enhances TCF7 expression to increase the Wnt7a/Wnt4/Wnt2b levels, which triggers the Wnt pathway. lncTCF7-mediated Wnt activation leads to the self-renewal maintenance and tumorigenic capacity of liver CSCs [19]. Unlike the mechanism of lncTCF7, lnc-*β*-Catm can strengthen *β*-catenin protein stability via posttranscriptional modification to activate the Wnt signaling pathway. lnc-*β*-Catm is located on chromosome 1q, which frequently occurs as copy-number amplification in liver cancer cells. lnc-*β*-Catm is highly expressed in liver CSCs and positively correlated with tumor aggressiveness and poor prognosis in liver cancer. lnc-*β*-Catm functions as a scaffold to associate *β*-catenin and the methyltransferase EZH2 in liver CSCs. Successively, EZH2 methylates *β*-catenin at Lys49, which abolishes its polyubiquitination and protects *β*-catenin from proteasomal degradation. *β*-Catenin subsequently forms a complex with TCF and acts as a cotranscriptional factor to initiate Wnt signaling. The lnc-*β*-Catm and EZH2-dependent *β*-catenin stabilization is required for oncosphere formation* in vitro* and increases tumorigenic cell frequency* in vivo *[47]. Additionally, lncRNA-DANCR has a novel mechanism to enhance liver CSC features by regulating *β*-catenin and stimulating the Wnt pathway. lncRNA-DANCR is dramatically overexpressed in liver CSCs and acts as an independent predictor for poor prognostic outcome in liver cancer. Instead of forming a complex with proteins, DANCR interacts with the *β*-catenin mRNA transcript within its 3′UTR that harbors miR-214 and miR-320a binding sites. The competitive occupation enables DANCR to block miRNA-mediated *β*-catenin depletion and raises the *β*-catenin reservoir to elevate its downstream AXIN2, NOTUM, and OAT levels. Through derepressing *β*-catenin and eliciting Wnt signaling, DANCR enhances the expansion and maintenance of liver CSCs [20].

### 3.2. lncRNAs Regulate Liver CSCs through the IL-6/STAT3 Cascade

Apart from the Wnt pathway, the IL-6/STAT3 signaling cascade plays important roles in regulating liver CSC maintenance as well. lncRNA DILC and lncSox4 have been established to control CSC features via the STAT3 pathway in liver cancer. lnc-DILC, which is downregulated in EpCAM^+^, CD24^+^, or OV6^+^ liver CSCs, has a tumor suppressive function. A stem cell signaling PCR array revealed that lnc-DILC can modulate the JAK2/STAT3 cascade. A gain in lnc-DILC expression decreases the activated phospho-STAT3 protein levels, attenuates STAT3 nuclear translocation, and represses the transcriptional activity of STAT3-responsive elements. Consistently, increased phospho-STAT3 is observed in lnc-DILC-silenced spheroid-formed xenografts. Mechanistic studies have revealed that lnc-DILC binds to the IL-6 promoter and inhibits its transcription, while IL-6 is a critical cytokine in the activation of the JAK2/STAT3 axis. Of note, IL-6 can be induced by the inflammatory factors, TNF-*α* and IL-1*β*, and the inflammatory microenvironment is a major aspect for liver cancer progression. In CSC spheroids, the downregulation of lnc-DILC also enhances TNF-*α* and IL-1*β*-induced IL-6 expression. These findings suggest that lnc-DILC can coordinate the crosstalk between inflammatory signaling and the autocrine IL-6/STAT3 pathway to promote liver CSC expansion [48]. Through comprehensive analysis of GSE datasets, lncSox4 upregulation has been identified in advanced liver cancer and poor prognostic samples. Further study has demonstrated that lncSox4 is primarily increased in the CD133^+^ liver cancer cell fraction and CSC spheroids, and lncSox4 is essential for liver CSC self-renewal and tumorigenic capacity. Mechanistically, lncSox4 interacts with STAT3 and recruits it to the Sox4 promoter region, inducing H3K4me3 and H3K27ac modification to drive the Sox4 promoter activation and augments Sox4 expression. The lncSox4/STAT3-dependent Sox4 expression exerts an indispensable function in sustaining liver CSC propagation, which may serve as an important target for CSC eradication [49].

### 3.3. lncRNAs Induce Liver CSCs via the Telomere-Related Pathway

Wu et al. found that lncRNA HULC and MALAT1 are dramatically upregulated in liver cancer cells. When MALAT1 is cooverexpressed with HULC in liver CSCs, the two lncRNAs can cooperate to promote liver CSC growth via the telomere repeat-binding factor 2 (TRF2). MALAT1 and HULC coexpression facilitates the TRF2 promoter and enhancer to form a loop, recruiting P300, RNA pol II, and CREPT into the loop, which enhances TRF2 expression and its phosphorylation and SUMOylation. Then, the excessive TRF2 forms a complex with HULC and MALAT1 on the telomeric region to protect telomeres from degradation. As a result, telomerase activity and microsatellite instability (MSI) are obviously induced in the liver CSCs [50]. CUDR is a novel lncRNA that can trigger the malignant transformation of hepatocyte-like cells through epigenetically remodeling TRF2, lncRNA HULC, and the *β*-catenin promoter structure to drive the expression of these oncogenes [51]. Furthermore, CUDR has been reported to be highly expressed in CD133^+^/CD44^+^/CD24^+^/EpCAM^+^ liver CSCs. Mechanistically, CUDR interacts with cyclin D1 to increase lncRNA H19 expression and enhance the association between TERT and TERC, thereby promoting telomerase activity and prolonging telomere length. Additionally, with the help of CTCF, the CUDR-cyclin D1 complex is recruited to the C-Myc gene promoter region to increase C-Myc expression. Synergistically, the excessive TERT and C-Myc expression account for liver cancer stem cell proliferation [52].

### 3.4. lncRNAs Affect Liver CSC Properties through Multiple Other Mechanisms

lncBRM is another upregulated lncRNA identified in the CD13^+^ CD133^+^ transcriptome microarray and is required for liver CSCs to maintain the self-renewal potential and initiate tumorigenicity. lncBRM can facilitate BRG1-embedded BAF complex formation and recruit the complex to the YAP1 promoter to trigger YAP1 transcription in a KLF4-dependent manner, thereby driving liver CSC properties [53]. lncRNA-mPvt1 is an oncofetal RNA that can promote stem cell-like properties in murine cells. Its human homologue lncRNA-hPVT1 is highly expressed in liver cancer cells and correlates with poor prognosis. lncRNA-PVT1 can interact with NOP2 and stabilize NOP2 from proteasomal degradation, which promotes malignant cell proliferation and self-renewal of spheroids [21]. Ding et al. identified a lncRNA, termed lncCAMTA1, that is overexpressed in CD13^+^ CD133^+^ CSCs and induces liver CSC proliferation. The lncCAMTA1 transcription orientation is antisense to the tumor suppressive gene CAMTA1, and their expression is negatively correlated in liver cancer samples. lncCAMTA1 physically binds to the CAMTA1 promoter and mediates a repressive chromatin structure to decrease CAMTA1 expression. The lncCAMTA1-dependent CAMTA1 downregulation accounts for liver CSC properties [54]. ICAM-1 is an established CSC marker in liver cancer [68]. LncICR is the ICAM-1-related lncRNA and is overexpressed in the ICAM-1^+^ liver CSC population. LncICR can form an RNA duplex with the ICAM-1 transcript via their complementary sequence, which increases the stability of ICAM-1 mRNA and augments its expression to maintain the liver CSC feature [55]. Lu et al. reported that the lncRNA HOTAIR can enhance liver cancer stem cell proliferation and malignant progression through downregulation of SETD2. The lncRNA HOTAIR can block the recruitment of the CREB-P300-RNA pol II complex to the SETD2 promoter to inhibit the expression and phosphorylation of SETD2, leading to the decreased formation of the hMSH6-H3k36me3-Skp2 complex to inhibit the DNA damage repair. In addition, the microsatellite instability (MSI) and abnormal expression of cell cycle related genes triggered by HOTAIR overexpression also contribute to the malignant growth of liver CSCs [56].

## 4. Perspective and Conclusion

miRNA and lncRNA are two major classes of ncRNAs. Previous studies have demonstrated that miRNAs and lncRNAs are stable in body fluids, and they can be easily and noninvasively accessible. Some of the miRNAs and lncRNAs have tissue- or disease-specific expression patterns. Therefore, miRNAs and lncRNAs have been recognized to be ideal biomarkers for early diagnosis, prognostic prediction, and therapeutic evaluation in cancer [69, 70]. For example, we have reported that low expression of miRNA-26a may predict poor prognosis and response to adjuvant INF-*α* treatment in liver cancer [71]. The prostate-specific lncRNA PCA3 has become the first FDA-approved lncRNA-based biomarker for prostate cancer diagnosis [72]. Additionally, several miRNA-targeted treatments have reached clinical trial, such as miRNA-34 mimics for treating cancer (phase I clinical trials) [73] and anti-miRs targeted miR-122 for remedying hepatitis (phase II clinical trials) [74]. Given the diverse regulatory mechanisms of ncRNAs in liver CSCs mentioned in this review, these dysregulated ncRNAs have great potential to be applied in diagnosis and prognosis. Furthermore, it may be feasible to target these aberrant ncRNAs to block or eradicate liver CSCs in cancer treatment.

**Figure 1:**
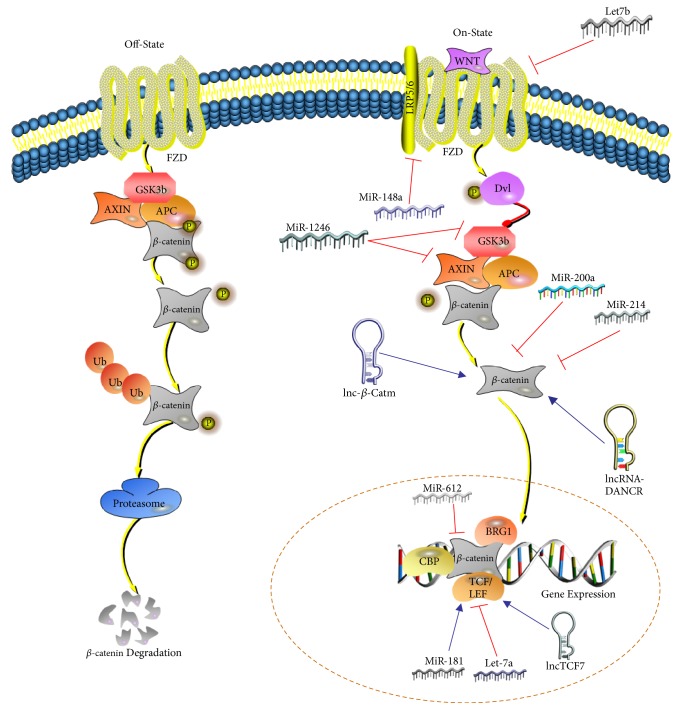
*The diverse regulatory mechanisms of ncRNAs on Wnt signaling pathway*. (A) Let7b can target and silence FZD4 expression. (B) miR-148a can inhibit the expression of ACVR1 to block the formation of Wnt-FZD-LRP5/6 receptor complex. (C) miR-1246 can directly suppress AXIN2 and GSK3*β* expression. (D) miR-200a can silence the expression of *β*-catenin. (E) miR-214 can deplete *β*-catenin expression. (F) miR-612 can indirectly decrease the nuclear accumulation of *β*-catenin. (G) miR-181 can enhance TCF activity by decreasing its inhibitor NLK. (H) Let-7a can directly silence TCF4. (I) lnc-*β*-Catm can lead to the methylation of *β*-catenin, enhancing the stability of *β*-catenin protein. (J) lncRNA-DANCR can bind to 3′-UTR of *β*-catenin to increase its expression by preventing *β*-catenin from depletion by miR-214 and miR-320a. (K) lnc-TCF7 can recruit SWI/SNF complex to TCF7 promoter and further elevate the activation of TCF7.

In this review, we emphasized the effects of ncRNAs on signaling pathways, finding that many miRNAs or lncRNAs control liver CSC properties by targeting different components of the Wnt/*β*-catenin pathway, like miR-148, miR-1246, miR-200a, lncRNA TCF-7, lncRNA *β*-Ctam, and so on (Figure 1). Recently, the Wnt/*β*-catenin axis has become an attractive therapeutic target because of its important functions in cancer. Several antibodies and small molecular inhibitors are undergoing preclinical or clinical trials, such as OMP-18R5 (antibody against FZD7), OMP54F28 (soluble FZD decoy receptor), and PRI-724 (inhibitor of TCF-CBP interaction) [75]. However, there are still no applicable candidates in clinical practice because of the serious side effects, as proper Wnt/*β*-catenin activity is essential to sustain normal cell survival. How to precisely regulate Wnt/*β*-catenin is a great conundrum. Targeting these regulatory miRNAs and lncRNAs may be an alternative approach to accomplish precise modulation of Wnt/*β*-catenin activation.

In conclusion, we overview the multiple functions and diverse mechanisms of miRNAs and lncRNAs in liver CSCs and highlight their potential clinical applications as novel diagnostic and prognostic biomarkers and therapeutic targets. Our review provides new insights to understand liver CSCs and delineates new clues to develop a ncRNA-based therapeutic strategy for liver cancer.

## Figures and Tables

**Table 1:** miRNAs participate in the regulation of liver CSCs.

miRNA	Expression	Liver CSC subtype	Targets	Reference
miR-181	↑	EpCAM^+^	CDX2, GATA6, NLK	[17]
miR-1246	↑	CD133^+^	AXIN2, GSK3*β*	[18]
Let7b	↓	CD24^+^CD133^+^	FZD4	[25]
miR-148a	↓	EpCAM^+^AFP^+^	ACVR1	[27]
miR-200a	↓	Side population	*β*-Catenin	[28]
miR-214	↓	EpCAM^+^	*β*-Catenin, EZH2	[29]
Let-7a	↓	Sphere formation	TCF4	[31]
miR-25	↑	CD133^+^	PTEN/PI3K/AKT/Bad	[32]
miRNA-21	↑	Side population	PTEN, RECK, PDCD4	[33]
miR-612	↓	EpCAM^+^CD133^+^	SP1/Nanog	[34]
miR-429	↑	EpCAM^+^	PBBP4/E2F1/OCT4	[35]
miR-145	↓	CD133^+^	Oct4	[36]
miRNA-200b	↓	CD13^+^CD24^+^	BMI1, CD13, CD24	[16]
miR-142-3p	↓	CD133^+^	CD133	[37]
miR-424, miR-222, miR-200b, let-7c	↓	*α*2*δ*1^+^	PBX3	[38]
miR-122	↓	CD133^+^	PDK4	[39]
miR-130b	↑	CD133^+^	TP53INP1	[40]
miR-155	↑	CD90^+^CD133^+^	TP53INP1	[41]
miR-152	↓	CD133^+^	KIT	[42]
miR-589-5p	↓	CD90^+^	MAP3K8	[43]
miR-150	↓	CD133^+^	c-Myb	[44]
miR-148b	↓	Side population	NRP1	[45]
miR-137	↓	CD133/44^+^EpCAM^+^	ANT2	[46]

**Table 2:** lncRNAs participate in the regulation of liver CSCs.

lncRNAs	Expression	Liver CSC subtype	Regulatory partners	Reference
lncRNATCF7	↑	CD133^+^CD13^+^	SWI/SNF complex	[19]
lnc-*β*-catm	↑	CD133^+^CD13^+^	*β*-catenin, EZH2	[47]
lncRNA-DANCR	↑	EpCAM^+^, CD90^+^	CTNNB1 mRNA	[20]
lnc-DILC	↓	EpCAM^+^ CD24^+^ OV6^+^	IL-6	[48]
LncSox4	↑	EpCAM^+^ CD133^+^	STAT3	[49]
HULC, MALAT1	↑	CD133^+^CD44^+^CD24^+^ EpCAM^+^	TRF2	[50]
lnc-CUDR	↑	CD133^+^CD44^+^CD24^+^ EpCAM^+^	CTCF	[51]
lnc-CUDR	↑	CD133^+^CD44^+^CD24^+^ EpCAM^+^	Cyclin D1	[52]
lncBRM	↑	CD133^+^CD13^+^	BRM	[53]
Lnc PVT1	↑	Sphere formation	NOP2	[21]
lncCAMTA1	↑	CD133^+^CD13^+^	CAMTA1	[54]
ICR	↑	ICAM-1^+^	ICAM-1 mRNA	[55]
HOTAIR	↑	CD133^+^CD44^+^CD24^+^ EpCAM^+^	CREB-P300-RNA polII complex	[56]
